# Am. Neurological Association

**Published:** 1875-08

**Authors:** 


[From the New York Medical Journal.]

### THE AMERICAN NEUROLOGICAL ASSOCIATION.

The first session of this Association was held in this city, June 3rd, 4th, and 5th, Dr. J. S. Jewell, of Chicago, occupying the chair. The following members were present: Drs. J. S. Jewell, Walter Hay, and H. M. Bannister, of Chicago; Dr. R. Bartholow, of Cincinnati; Dr. J. K. Bauduy, of St. Louis; Dr. M. Burnett, of Knoxville; Dr. E. R. Hun, Jr., of Albany; Drs. E. H. Clarke, R. T. Edes, J. J. Putnam, S. G. Webber, and F. D. Lincoln, of Boston; Drs. S. Weir Mitchell, William Pepper, and H. C. Wood, Jr., of Philadelphia; Drs. J. Van Bibber, and F. T. Miles, of Baltimore; Dr. H. D. Schmidt, of New Orleans; Dr. F. D. Lente, of Cold Spring, N.Y.; Dr. J. C. Shaw, of Brooklyn; Drs. W. A. Hammond, Meredith Clymer, E. C. Seguin, T. M. B. Cross, J. J. Mason, F. K. Kinnicutt, A. D. Rockwell, D. B. St. John Roosa, A. McLane Hamilton, E. G. Loring, J. C. Dalton, J. W. S. Arnold, N. B. Emerson, T. A. McBride, and J. S. Lombard, of New York.

On motion of Dr. McBride, the following resolution was adopted:

"It being the sense of this Association that its proceedings should be reserved for the profession and the medical press, be it

"*Resolved*, That reporters from non-medical journals be excluded from the meetings of the Association; and that any information requested by the press be furnished by the Secretary, under the guidance of the officers of the Association."

The Committee on Organization, through Dr. Meredith Clymer, reported the subjoined Constitution and By-Laws, which were adopted:

### CONSTITUTION.

I. This Association shall be named and known as "The American Neurological Association."

II. It is established to promote the study of neurological science in all its departments.

III. There shall be two sorts of members, namely, active members—not exceeding at any one time fifty in number, and who shall be at the time of their election residents of the United States—and foreign associate members, not exceeding at any one time twenty-five in number, and who shall be non-residents. Active and foreign associate members shall be elected by ballot on the recommendation of the Council, on one day's previous notice of such ballot, by a majority of all the members present:

*Provided*, That no one shall be eligible for active membership unless he has previously submitted a paper on some subject connected with neurological science, which paper shall be referred to the Council for examination and report. Active members only shall be entitled to vote at any meeting, or be eligible to any office.

IV. The officers of the Association shall be a President, two Vice-Presidents, a Corresponding Secretary, a Recording Secretary, who shall perform the duties of Treasurer, and a Curator. They shall be nominated by a Committee of Nomination of five members, appointed by the President on the first day of the annual session, and who shall report on the day following, immediately after which the election shall take place. The election shall be by ballot, and the person who shall have the greatest number of votes shall be declared elected to the office for which he may be a candidate.

In case of a vacancy occurring in any office between the dates of the annual election, it shall be filled by the Council until the next annual election.

The officers shall enter upon their duties immediately after the organization of the annual session next after their election, and shall hold office for one year:

*Provided*, That the officers of the first session shall be elected immediately after the organization of the Association, and shall hold their offices until the election at the second annual session.

V. The Council shall consist of the officers of the Association, shall manage the affairs of the Association, subject to the Constitution and By-Laws, and shall report to the Association at large at each annual session.

VI. The annual session of the Association shall be held on the first Monday in June, in each year, and at such place as shall be designated by the Association at the previous annual session, and shall continue for three days, unless the time be extended by a vote of the Association.

VII. This Constitution may be amended by a two-thirds vote of all the members present, at any annual session, provided that notice of said proposed amendment in writing be given at the annual session immediately preceding.

BY-LAWS.

1. Each and every member of the Association shall pay annually to the Recording Secretary the sum of five dollars.

No member who shall be in arrears for one year shall be entitled to vote, or be eligible to any office in the Association.

2. The officers of the Association shall discharge the duties belonging to their respective offices. The President shall be *ex-officio* chairman of the Council.

3. The Council shall meet as often as the business of the Association may require. They shall keep a record of their proceedings, which shall be read at the annual session of the Association. They shall not have power to make the Association liable for any debts exceeding in total the sum of one hundred dollars in the course of any one year, unless specially authorized to do so by a recorded vote of the Association.

4. The order of business at each meeting of the Association shall be as follows :

5. The titles of all papers to be read at any annual session shall be forwarded to the Corresponding Secretary not later than one month before the first day of the session. All papers that may be read before the Association and accepted shall become the property of the Association, and their publication shall be under the control of the Council. All publications of the meetings of the Association shall be under the direction of the Council.

6. These By-Laws, or any one or more of them, may be amended, or repealed, or suspended, by a two-thirds vote of all the members present at any meeting during an annual session, provided notice in writing of any proposed amendment or repeal has been given at the meeting immediately preceding the one at which the motion is made and the vote taken.

The following report of the nominating committee was unanimously adopted :

*For President*—Dr. S. Weir Mitchell, of Philadelphia.

*For Vice-Presidents*—Dr. J. S. Jewell, of Chicago; Dr. E. H. Clarke, of Boston.

*For Corresponding Secretary*—Dr. J. J. Mason, of New York.

*For Curator*—Dr. J. W. S. Arnold, of New York.

*For Recording Secretary and Treasurer*—Dr. E. C. Seguin, of New York.

A communication was subsequently received from Dr. S. Weir Mitchell, containing his resignation of the presidency, and Dr. Jewell was elected President in his place, Dr. E. H. Clarke being made first Vice-President, and Dr. F. D. Miles, second Vice-President.

A paper on the study of myelitis, by Dr. Webber, of Boston, was read and discussed.

Dr. Miles reported a case of spinal paralysis, with partial recovery.

Dr. Hammond presented a case of athetosis.

Dr. Van Bibber read a paper on the treatment of paralyzed muscles by elastic relaxation.

Dr. Putnam reported an interesting case of injury of the brachial plexus.

Dr. Van Bibber reported also a case of coincident nerve-injury and cutaneous eruption, which called forth remarks on similar cases by several gentlemen.

Dr. Rockwell read a paper on electro-medicine with reference to its physiological and therapeutical relations to the nervous system.

Dr. Putnam read a paper on a case of circumscribed analgesia of the skin after typhoid fever.

Dr. Hamilton exhibited a new and ingenious dynamometer, consisting essentially of a rubber ball, and a glass tube to show and register the degree of pressure exerted on the ball.

Dr. Hun presented a specimen of fracture of the odontoid process.

Dr. Lente read a paper on neuralgia and other neuroses arising from cicatrices in the scalp.

Dr. Hammond presented a paper on pigmentary deposits on the brain as the result of malarial poisoning.

Dr. Hay offered a specimen of hemiplegia, with clot on the same side.

Dr. Hammond nominated for membership Drs. S. Oakley Vanderpoel, T. Edwards Clark, and Clinton Wagner, of New York.

Dr. Mason nominated Drs. J. P. Gray, of Utica, and D. H. Kitchen, of New York.

Dr. E. C. Seguin nominated Dr. G. M. Beard, of New York.

Dr. Hammond offered the following:

*Resolved*, That, as the number of active members of this Association is limited to fifty, it is inexpedient to elect to membership superintendents of lunatic asylums, but that in this action the Association does not wish to depreciate the labors of these gentlemen, and is further influenced in this action by the fact that there is already an association composed exclusively of superintendents of such asylums.

After discussion, the matter was referred to the Council, with instructions to report at the next session of the Association.

Drs. Clymer, Hammond, and Hamilton, were appointed a Committee of Arrangements to act in conjunction with the Council for the session of next year. It was decided to hold the next session of the Association in this city, on the first Wednesday in June, 1876.

